# Short- and long-term outcomes of adrenalectomy for primary aldosteronism in a single UK center: rear-mirror view

**DOI:** 10.1007/s42000-024-00613-3

**Published:** 2024-11-18

**Authors:** Tarek Abdel-Aziz, Alaa Abdelsalam, Teng-Teng Chung, Umasuthan Srirangalingam, Steven Hurel, Gerard Conway, Stephanie E. Baldeweg, Tom R. Kurzawinski

**Affiliations:** 1 Centre for Endocrine Surgery, University College London Hospitals NHS Foundation Trust, 250 Euston Road, London, NW1 2PG UK; 2 Faculty of Medicine, Alexandria University, Alexandria, Egypt; 3 Endocrinology department, University College London Hospitals NHS Foundation Trust, London, UK

**Keywords:** Primary aldosteronism, Adrenalectomy, Conn’s syndrome, Biochemical cure

## Abstract

**Purpose:**

Primary aldosteronism (PA), which is the commonest cause of secondary hypertension, can be cured by unilateral adrenalectomy. We report the short-and long-term outcomes after adrenalectomy performed at a single UK center over a period of 24 years.

**Methods:**

Retrospective analysis of *biochemical* (potassium, aldosterone, renin, and ARR) *radiological* (CT/MRI, AVS, and nuclear scans), and *clinical* (surgical complications, blood pressure, and number of antihypertensive medications) short-and long-terms outcomes in patients who underwent adrenalectomy for PA between 1998 and 2021. Standardized PASO and Clavien-Dindo criteria to assess biochemical, clinical, and surgical outcomes were used.

**Results:**

A total of 82 patients were treated via adrenalectomy for PA over a 24-year period. Short-term follow-up data (within 3 months after surgery) was available for all 82 patients (M45, F37, mean age 51.7 years): 24 of them were followed up for at least 60 months (range 60 to 72 months) and 77 (93.9%) patients had laparoscopic surgery (one conversion). Seven patients had postoperative complications classified as Clavien-Dindo II (4), IIIa(1) and IVa(2). Median LOS was 2.5 days (1–12). Complete and partial clinical success was achieved in 29 and 58.3% and 41.7 and 45.8% of patients in the short and the long term, respectively. Clinical benefit was observed in 88% of patients. Complete biochemical success was achieved in 95.8% of patients in the short and the long term.

**Conclusion:**

Unilateral adrenalectomy in patients with PA showed clinical benefit in 88% and achieved biochemical cure in almost all of them. Our data suggest that these benefits persisted for at least 5 years.

## Introduction

Primary hyperaldosteronism (PA) is the commonest cause of secondary hypertension and can be successfully treated by adrenalectomy if excessive secretion of aldosterone is confirmed to be from a unilateral source [1, 2]. Estimates of the prevalence of PA have varied considerably over the last 70 years, but recent data suggest that it affects 3.2 to 12.7% of patients in primary care, while 1 to 29.8% are referred to hypertension units [3, 4]. The incidence of PA rises with the severity of hypertension, from 2% in patients with grade 1 hypertension to 20% in those with resistant hypertension [4].

Patients with PA have an increased risk of myocardial infarction, stroke, atrial fibrillation, and higher prevalence of metabolic syndrome in comparison to patients with essential hypertension [3]. Recent meta-analyses have shown that patients with PA treated with adrenalectomy had better outcomes in comparison to patients treated with medications alone [5]. This is because surgery can not only restore normal blood pressure and obviate the need for antihypertensive medications, but also, by removing the source of aldosterone over-secretion, can normalize the renin-angiotensin-aldosterone system and correct concomitant abnormalities of hypokalemia, alkalosis, and fibrosis in multiple organs, such as the heart, kidney, and pancreas, caused by high levels of aldosterone [6–8].

The introduction of minimally invasive techniques had a huge positive impact on outcomes after adrenalectomy in reducing postoperative pain, length of hospital stay, and improved cosmesis. Cure rates after adrenalectomy for PA range from 32 to 42% at 6 months after surgery, but data on long-term outcomes are relatively scarce and often consist of small cohort studies [9–13]. It is not clear whether short-term benefits of adrenalectomy persist in the longer term and whether clinical and biochemical cure go hand in hand or have a separate course [14].

Interest in PA has increased in recent years and research into this condition has experienced what can be called a “Renaissance period,” leading to the development of new functional imaging and adrenal ablative techniques as an alternative to surgical resection [15–18]. Understanding the value of novel and emerging diagnostic and therapeutic techniques and their place in the context of current practice can be informed by reviewing outcome data achieved in the preceding decades, which were characterized by a relatively stable diagnostic and therapeutic ‘best practice’ pathway.

Our study aims to evaluate the “real-life experience” of diagnosing and treating patients with PA in a single tertiary referral center in the UK over 24 years, with a focus on the short-and long-term outcomes of adrenalectomy for PA and the introduction of the laparoscopic approach as a treatment for this condition.

## Materials and methods

Retrospective analysis of data from a prospectively maintained database of patients with PA who underwent surgery between 1st January 1998 and 31st December 2022 at University College London Hospital, a tertiary referral center for endocrinology and endocrine surgery.

The clinical pathway for screening, diagnosis, and stratification for adrenalectomy varied through the 24 years and was based on concurrent recommendations, published evidence, changing local expertise, and best practice at the time. Throughout this period, diagnosis of PA in our center was based on the presence of hypertension and hypokalemia, high aldosterone level, suppressed renin activity, and aldosterone and renin ratio (ARR) above 850. Currently, a confirmatory test (for example, the saline suppression test) is recommended if the aldosterone is < 550pmol/L but in the upper third of the normal range or the plasma renin is non-suppressed, unless the patient presents with spontaneous hypokalemia, plasma renin below detection levels, and aldosterone concentration more than 550 pmol/L.

Stratification for adrenalectomy, once a diagnosis of PA was confirmed, included cross-sectional imaging of adrenals with CT or MRI [2]. Adrenal venous sampling (AVS) and/or later ^11^C metomidate PET-CT was performed if imaging showed bilateral adrenal nodules [2]. Surgery was recommended after discussion in the multidisciplinary team meeting (endocrinologist, endocrine surgeon, biochemists, and radiologist).

At the very beginning of this study, laparoscopic adrenalectomy was introduced in our center and the transperitoneal lateral technique was used to perform laparoscopic adrenalectomy using three ports on the left and four ports on the right side.

Patients included in the study were characterized preoperatively by their demographics (age, sex, and ethnicity) clinical presentation (blood pressure), number of antihypertensive medications, and biochemical tests (potassium, aldosterone, renin, and ARR).

The number and types of investigations (CT, MRI, AVS, and metomidate) were used to stratify for surgery and the size of the adrenal nodules on cross-sectional imaging was recorded. The process of stratification was presented as lateralization to the left or the right reached as a consensus between cross-sectional imaging, AVS, and nuclear medicine imaging.

Data on postoperative surgical outcomes included conversion rate to open adrenalectomy, postoperative complications (Clavien-Dindo) [19], and length of hospital stay.

Postoperative outcomes were assessed within 3 months of surgery (short-term) and at least 60 months (long-term) after surgery. Data collected were blood pressure, number of antihypertensive medications, plasma concentration of potassium, aldosterone, renin, and ARR. Surgical outcomes were categorized using the PASO criteria for clinical outcomes of complete, partial, or absent success for both clinical and biochemical cure which reflected remission, improvement, or persistence of disease [6].

### Statistical analysis of the data

The data were analyzed using IBM SPSS software package version 20.0. (Armonk, NY, USA: IBM Corp). Qualitative data were reported using absolute and relative (%) frequencies. The Kolmogorov-Smirnov test and the Shapiro-Wilk test were used to verify the normality of distribution. Quantitative data were described using range (minimum and maximum), mean, standard deviation, and median. The paired t-test was assessed for comparison between two periods for normally distributed quantitative variables, while the Wilcoxon signed-rank test was assessed for comparison between two periods for abnormally distributed quantitative variables. ANOVA with repeated measures was employed for normally distributed quantitative variables to compare more than two periods or stages, while the Friedman test was used for abnormally distributed quantitative variables to compare between more than two periods or stages and the post hoc test (adjusted Bonferroni correction and Dunn’s) for pairwise comparisons. The marginal homogeneity test was used to analyze the significance between the different stages. A two-tailed *p*-value < 0.05 was considered statistically significant.

## Results

### Demographics, presentation, biochemistry, and stratification for surgery for the whole cohort of 82 patients

Eighty-two patients diagnosed with PA were included in the study. Forty-five (54.9%) of them were male, 37 (45.1%) were female, their mean age being 51.7 years (16.2–75.7). Forty-three (52.4%) patients were white, 18 (21.9%) African/Caribbean, six (7.3%) mixed white/black, nine (10.9%) Asian, and in six the patients’ ethnicity was unknown (7.3%).

Seventy-seven patients (93.9%) presented with hypertension and 61 (74.4%) had hypokalemia. Three (3.7%) patients had adrenal incidentalomas which led to the diagnosis of PA. Patients’ mean (SD) systolic and diastolic blood pressure was 165.1mmHg (23.7) and 93mmHG (14.9), respectively, and mean (SD) potassium level was 3.1mmol/L (0.7). Median aldosterone level was 932 pmol/L (450–2900), renin 0.3 nmolL/H (0.1–2.7), and ARR 2824 (2444–9233).

Fifty-five (67%) patients underwent a CT scan and 10 (12.1%) MRI only, while 17 (20%) had both. In total, 46 patients (50%) were stratified for surgery following biochemical assessment and imaging with CT or MRI only. The median size of adrenal nodules on cross-sectional imaging was 1.8 cm (range 0.4–7.5).

Twenty-six (31.7%) patients had AVS in addition to imaging, with an 84.8% success rate, seven (8.5%) had both AVS and ^11^C metomidate PET-CT, while three (3.6%) had an ^11^C metomidate PET-CT scan alone.

### Surgery, histology, and surgical outcomes for the whole cohort of 82 patients

Laparoscopic adrenalectomy was performed in 77 patients (93.9%) and four (4.9%) had open procedures. Laparoscopic adrenalectomy was converted to open surgery in one patient due to bleeding (1.2%). In total, 45 patients had right (54.8%) and 37 left adrenalectomy (45.1%).

Four patients had postoperative infections treated with antibiotics (Clavien-Dindo II) and one (1.2%) had infected fluid collection in the adrenal bed which required antibiotics and percutaneous drainage (Clavien-Dindo IIIa). Two patients (2.4%), in whom spironolactone had not been discontinued, developed hyperkalemia with an acute kidney injury (Clavien-Dindo IVa); both were treated conservatively and fully recovered. Median hospital stay was 2.5 days (1–12).

Histopathology reported 71 adenomas, six cases of multinodular hyperplasia, and five adenomas with nodular hyperplasia. The mean (SD) dimension of adenomason histology examination was 2.3 (1.6) cm (range 0.6–7.5 cm).

### Short-term biochemical and clinical outcomes at 3 months for 82 patients (whole cohort) (Table 1)

There was statistically significant improvement in mean systolic and diastolic blood pressure postoperatively, from 165 mmHg to 131 mmHg and from 93 mmHg to 82 mmHg, respectively (p- value < 0.001). All patients who presented with hypokalemia had normal potassium postoperatively, with mean potassium rising from 3.1 to 4.5 mmol/L.

**Table 1:** Pre- and 3 months postoperative blood pressure and potassium levels in the 82 patients (whole cohort) following adrenalectomy for PA

	Preoperative	Postoperative	*p*
Potassium (mmol/L)			
Low < 3.5	61 (74.4%)	0	< 0.001
Normal (3.5–5.3)	21 (25.6)	76 (92.7%)	
High (> 5.3)	0	6 (7.3%)	
Mean ± SD	3.1 ± 0.7	4.5 ± 0.6	
Blood pressure (mmHg)			
Systolic			
Mean ± SD	165.1 ± 23.7	131.7 ± 16.1	< 0.001^*^
Diastolic			
Mean ± SD	93 ± 14.9	82 ± 10.7	< 0.001^*^

SD: standard deviation, *t*: paired t-test

*p*: *p* value for comparing between preoperative and postoperative

### Short-and long-term clinical and biochemical outcomes at 5 years in the cohort of 24 patients (Table 2, 3, 4 and 5)

Our results showed that the subgroup of 24 patients with at least 5 years’ follow-up, similarly to the whole cohort of 82 patients, gained significant clinical and biochemical benefit from adrenalectomy in a short term and that this effect was sustained at 5 years.

Preoperative hypokalemia was cured in all, aldosterone levels were significantly reduced, and renin became unsuppressed, and, because of these changes, ARR normalized (*P*-value < 0.001) (Table 2). Interestingly and very importantly, biochemical cure was sustained 5 years later as indicated by lack of statistical significance when comparing short- and long-term outcomes (*P*-value 0.8).

**Table 2:** Pre-, 3 months, and 5 years postoperative biochemical outcomes in the 24 patients following adrenalectomy for PA

Lab	Preoperative	Postoperative	Follow-up	*p*
**Potassium**				
Median (min.– max.)	3.2 (2.4–4.9)	4.8 (3.6–5.9)	4.6 (3.4–5.5)	< 0.001^*^
Mean ± SD.	3.3 ± 0.6	4.7 ± 0.6	4.6 ± 0.5	
**Sig. bet. periods**	*p*_1_ < 0.001^*^, p_2_ < 0.001^*^, p_3_ = 0.752			
**Aldosterone (pmol/L)**				
Median (min.– max.)	932 (450–2900)	115 (60–723)	205 (70–1480)	< 0.001^*^
Mean ± SD.	1206.1 ± 729.9	161.8 ± 137	259.4 ± 279.5	
**Sig. bet. periods**	*p*_1_ < 0.001^*^, p_2_ < 0.001^*^, p_3_ = 0.112			
**Renin (nmol/L/H)**				
Median (min.– max.)	0.3 (0.1–2.7)	1 (0.2–3.9)	1.5 (0.4–8.4)	0.001^*^
Mean ± SD.	0.8 ± 0.9	1.2 ± 0.8	1.8 ± 1.5	
**Sig. bet. periods**	*p*_1_ = 0.036^*^, p_2_ < 0.001^*^, p_3_ = 0.149			
**ARR**				< 0.001^*^
Median (min.– max.)	2824.5(244.4–9233)	104.5(23–2410)	127.5(23.8–510)	
Mean ± SD.	3413.1 ± 2873.4	280.9 ± 512.8	169.8 ± 114.5	
**Sig. bet. periods**	*p*_1_ < 0.001^*^, p_2_ < 0.001^*^, p_3_ = 0.885			

SD: standard deviation, Fr: Friedman test, Sig. bet. periods was done conducting the post hoc test (Dunn’s)

*p*: *p* value for comparing between the different studied periods

*p*_1_: *p* value for comparing between preoperative and 3 months

*p*_2_: *p* value for comparing between preoperative and 5 years

*p*_3_: *p* value for comparing between postoperative and 5 years

Similarly, patterns of preoperative short-and long-term data suggest that adrenalectomy significantly reduced both systolic and diastolic blood pressure in the short term and that this benefit was sustained in the long term (*P*-value < 0.001 and 1, respectively) (Table 3).

**Table 3:** Pre-, 3 months, and 5 years postoperative clinical outcomes in the 24 patients following adrenalectomy for PA

Blood pressure	Preoperative	3 months	5 years	*p*
**Systolic (mmHg)**				
Mean ± SD.	158.4 ± 25	131.8 ± 14.2	132.5 ± 11.2	< 0.001^*^
**Sig. bet. periods**	*p*_1_ < 0.001^*^, p_2_ < 0.001^*^, p_3_ = 1.000			
**Diastolic (mmHg)**				
Mean ± SD.	93 ± 14.9	82 ± 10.7	81 ± 9.4	< 0.001^*^
**Sig. bet. periods**	*p*_1_ = 0.008^*^, p_2_ = 0.003^*^, p_3_ = 1.000			

SD: standard deviation, F: F test (ANOVA) with repeated measures, Sig. bet. periods was conducted the using post hoc test (adjusted Bonferroni)

*p*: *p* value for comparing between the different studied periods

*p*_1_: *p* value for comparing between preoperative and 3 months

*p*_2_: *p* value for comparing between preoperative and 5 years

*p*_3_: *p* value for comparing between postoperative and 5 years

The number of antihypertensive medications prescribed before and after surgery, an important measure of clinical success of treatment, is shown in Tables 4 and 5. Before surgery, 1/3 of the patients required 1–2 medications, with the majority (2/3) needing 3–6 antihypertensives to control their blood pressure. Within 3 months of adrenalectomy, about 1/3 did not take any and half were able to reduce medications and had blood pressure controlled with 1–2 medications. Only two patients (10%) needed three medications, none requiring more than that. These differences between the number of tablets taken before and after surgery were statistically significant. At 5 years, more patients were able to stop or reduce their medications, with some carrying on with the same tablets and only one patient having to increase medications.

**Table 4:** Pre-, 3 months, and 5 years postoperative number of antihypertensive medication outcomes in the cohort of 24 patients

Number of medications	Preoperative	3 months	5 years	*p*
0	0 (0%)	7 (29.2%)	10 (41.7%)	< 0.001^*^
1	5 (20.8%)	6 (25%)	10 (41.7%)	
2	4 (16.7%)	9 (37.5%)	2 (8.3%)	
3	8 (33.3%)	2 (8.3%)	2 (8.3%)	
4	4 (16.7%)	0 (0%)	0 (0%)	
5	2 (8.3%)	0 (0%)	0 (0%)	
6	1 (4.2%)	0 (0%)	0 (0%)	
**Sig. bet. periods**	*p*_1_ < 0.001^*^, p_2_ < 0.001^*^, p_3_ = 0.312			

Fr: Friedman test, Sig. bet. periods was conducted using the post hoc test (Dunn’s)

*p*: *p* value for comparing between the different studied periods

*p*_1_: *p* value for comparing between preoperative and 3 months

*p*_2_: *p* value for comparing between preoperative and 5 years

*p*_3_: *p* value for comparing between postoperative and 5 years

**Table 5:** Comparison between the two studied periods according to medication in the cohort of 24 patients

Medication	3 months	5 years	*p*
Stopped Reduced	7 (29.2%) 14 (58.3%)	10 (41.7%) 5 (20.8%)	0.008^*^
Same	3 (12.5%)	8 (33.3%)	
Increased	0 (0%)	1 (4.2%)	

MH: marginal homogeneity test

*p*: *p* value for comparing between postoperative and 5 years

Twenty-one patients (87.55%) had clinically benefited from surgery and showed, both in the short and the long term, complete or partial clinical success according to the PASO criteria (Table 6). More patients were classified as completely cured at 5 years as three patients who were classified as partial success in the short term did not require antihypertensives at 5 years.

**Table 6:** Comparison between short-and long-term outcomes according to PASO criteria in the cohort of 24 patients

PASO group	3 months	5 years
Complete clinical success	7 (29.2%)	10 (41.7%)
Partial clinical success	14 (58.3%)	11 (45.8%)
Absent clinical success	3 (12.5%)	3 (12.5%)
Complete biochemical success	23 (95.8%)	23 (95.8%)
Partial biochemical success	0 (0%)	0 (0%)
Absent biochemical success	1 (4.2%)	1 (4.2%)

Biochemical success was unequivocal, with all but one case being classified as a complete biochemical success. Only one patient, a 23-year-old woman with a 2.4 cm adrenal adenoma, was not cured biochemically in the short term.

In the long term review, the blood pressure was well controlled on 100 mg spironolactone.

## Discussion

The rapid advances being made in the field of PA come mostly from basic scientific research in molecular biology and genetics and, more recently, also in the form of new ideas about functional imaging and novel therapeutic interventions for this condition [1, 16–18, 20]. These concepts are now moving from the bench to the bedside of our patients and are being assessed prospectively in clinical trials, although it will be some time before their impact on clinical practice is known [16, 17]. However, while speeding down the lane of progress, we should not lose sight of milestones already reached as they will help us to judge the shortcomings of new interventions. It is expected that our study, which presents the short- and long-term outcomes of adrenalectomy in patients with PA in a single center over the last 24 years, may contribute to the current debate concerning PA by providing such a rear-mirror view.

*Firstly*, one might consider that only 82 adrenalectomies for PA performed over a 24-year period in a tertiary center represents a relatively limited number of cases given the predictedprevalence. To our knowledge, this low number of adrenalectomies for PA is not peculiar to our center but is a common experience occurring worldwide [21]. As observed by J. W. Conn in 1955, around 10% of hypertensive patients will have PA; however, the reality is that we are diagnosing and operating on only about 1% of patients with this condition. Clearly, case identification needs to increase, which will be achieved by improving awareness amongst patients and doctors but also by simplifying the current diagnostic pathways.

*Secondly*, our paper describes a pragmatic approach to localization for surgical management, which procedure was used during the study period. The pivotal investigation in our localization strategy was CT or MRI to identify adrenal nodules. Patients with conclusive biochemistry and clear unilateral nodules were offered surgery, especially if young. Patients with bilateral nodules or who were over 35 years of age were tested further with a combination of AVS and functional imaging. Since this approach did not offer AVS routinely but selectively, it is interesting to compare outcomes of other studies where AVS was utilized more comprehensively.

Overall cure rate in such studies published in the past, which took into account biochemical and clinical outcomes, have typically been reported to vary between 32 and 47% [1, 6, 14, 21, 22]. A more recent PASO study showed complete, partial, or absent biochemical success in 94%, 4%, 2%, respectively, and complete, partial, or absent clinical success in 37%, 47%, 16% of patients, respectively [6]. This study concludes that biochemical and clinical benefit was observed in 98 and 84% of patients. In a MATCH study, complete biochemical and clinical success was achieved in 88and 31% of patients, respectively [16].

In our study, outcomes at 3 months for the whole cohort of 82 patients showed that hypokalemia was cured in all patients and statistically significant reduction in blood pressure was achieved. Short-term results in 24 patients with long-term follow-up available showed that all but one patient achieved a statistically significant decrease in aldosterone levels, normalization of ARR, improvement in blood pressure, and reduction of medications. At 3 months, complete biochemical success was achieved in 95.8% and complete and partial clinical success in 29.2% and 58.35%, suggesting clinical benefit in 87.55% of our patients. What is remarkable is that these benefits were sustained or had even improved at 5 years and only patients who were not cured at 3 months remained so at 5 years. All patients cured biochemically and clinically in the short term remained cured at 5 years. Clinical benefit in the long term remained the same as in the short term (87.5%), but more patients achieved a complete cure (41.7%), perhaps reflecting the need for long-term follow-up as blood pressure improves and medications are reduced.

What are the possible explanations that our outcomes are not inferior to results from series where routine AVS was used? Perhaps it is the fact that AVS is a less than perfect “gold standard” in predicting cure. The SPARTACUS trial found no difference in cure rate whether AVS was used or not, although it was criticized for its design and for being underpowered [23]. A recently published MATCH study showed that AVS predicted biochemical and clinical cure with 63.3 and 61.5% accuracy [16]. Metomidate was just marginally accurate with an accuracy of 72.7 and 65.4%. CT and AVS discrepancy is known to vary from 30 to 40%, but does this always mean that patients who have not met strict criteria for lateralization (LI > 4) and are not offered surgery would not have benefited? Published evidence on unilateral adrenalectomy in patients with known bilateral PA suggest otherwise as they reveal biochemical and clinical benefit from what is known as the debulking effect; thus, some of our patients might have benefited from it [24].

It is also likely that our good outcomes were the result of stratification based on the presence of radiologically obvious and sizable adrenal nodules. Recent studies have revealed that patients with classic histopathology who form adenomas are more likely to have unilateral disease and that their cases are associated with better chances of cure if adrenalectomy is performed [25, 26]. Non-classical histopathology associated with hyperplasia and formation of APMs (formerly known as APCC), which are not visible on CT, tend to be bilateral and are less likely to be cured by unilateral adrenalectomy [25, 26].

The fact that in our series, 76 (93%) patients had sizable adenomas and only six had hyperplasia would suggest that our population was artificially enriched by this kind of selection, that is, in choosing patients who were more likely to be cured. Future studies into somatic mutations responsible for formation of either radiologically detectable or “invisible” adenomas and APMs will help to clarify the etiology of these distinct forms of PA.

It is also known that results are better in women and younger people; however, sex and age distribution in our study does not explain this and neither does ethnic mix.

*Thirdly*, our results strongly confirm that unilateral adrenalectomy is currently the gold standard therapy for patients with PA. There is consensus that, in comparison to medical therapy, adrenalectomy offers better management of hypertension, hypokalemia, and left ventricular hypertrophy [21]. The present series concurred with the introduction of laparoscopic adrenalectomy in our center, one of the first in the UK. Some open adrenalectomies were performed during the transitional period, but most operations were laparoscopic procedures with only one conversion. The low complication rate confirmed the safe profile of the laparoscopic approach and the associated short post-operative stay (even shorter recently) contributes to its cost-effectiveness [27, 28]. New approaches, such as partial adrenalectomy or ablative techniques using extreme temperatures causing freezing or boiling of adrenal tissue, carry significant risk of not achieving a cure in the first place and/or resulting in early recurrence of PA [18].

Ablative approaches also have a very high cost (use of e.g., catheters and probes), take a longtime, are associated with increase in radiation, require general anesthesia, and are not currently performed as day-case surgery; hence, they might not be cost-effective at all.

We acknowledge that the present study has certain limitations. The retrospective design could have affected the accuracy of the collected data, especially as it was carried out over a long period of time. Availability of long-term followup data in only one-third of our patients is also a limitation.

In summary, our single center results showed that good biochemical and clinical outcomes in patients with PA have been achieved in the past 24-year period, this coinciding with the switch to laparoscopic adrenalectomy. These good results were almost certainly due to a highly selected population of patients with PA, with sizable and radiologically visible adrenal nodules, who were more likely to benefit from adrenalectomy, even if AVS was used selectively rather than routinely. Future developments should focus on identifying the vast majority of subjects with PA who are not currently diagnosed or offered surgery. The current existing pathway for screening, confirming, and localizing disease is complex, time-consuming, and inconvenient. AVS, which is now 60 years old and one of the oldest procedures still performed by interventional radiologists, is unlikely to be perfected any further and new solutions are needed.

Novel molecular imaging of the adrenal glands could provide a solution with the potential to reshape the scientific and clinical landscape of primary aldosteronism. Cholesterol imaging used in the past had very limited accuracy and metomidate and its derivatives have wellknown limitations due to their non-selectivity for enzymes involved in the aldosterone pathway and difficulty with isotope production^15^. However, recent research involving tracers based on highly selective aldosterone synthase blockers showed that such tracers could reliably detect not only adenomas but also aldosterone-producing microadenomas (APMs) in human adrenals both in vitro and in vivo^17^. Possibly, imaging based on this technology could shorten the diagnostic pathway by making a confirmatory step unnecessary and merge subtyping investigations into a single scan. Such a transformation of the PA diagnostic pathway should improve the accuracy of the diagnosis and the efficiency of stratification for surgery and would be beneficial to patients in the future.
